# A Germline Mutation in the *POT1* Gene Is a Candidate for Familial Non-Medullary Thyroid Cancer

**DOI:** 10.3390/cancers12061441

**Published:** 2020-06-01

**Authors:** Aayushi Srivastava, Beiping Miao, Diamanto Skopelitou, Varun Kumar, Abhishek Kumar, Nagarajan Paramasivam, Elena Bonora, Kari Hemminki, Asta Försti, Obul Reddy Bandapalli

**Affiliations:** 1 Division of Molecular Genetic Epidemiology, German Cancer Research Center (DKFZ), 69120 Heidelberg, Germany; a.srivastava@kitz-heidelberg.de (A.S.); d.skopelitou@kitz-heidelberg.de (D.S.); abhishek@ibioinformatics.org (A.K.); k.hemminki@dkfz.de (K.H.); a.foersti@kitz-heidelberg.de (A.F.); 2 Hopp Children’s Cancer Center (KiTZ), 69120 Heidelberg, Germany; b.miao@kitz-heidelberg.de; 3 Division of Pediatric Neurooncology, German Cancer Research Center (DKFZ), German Cancer Consortium (DKTK), 69120 Heidelberg, Germany; 4 Medical Faculty, Heidelberg University, 69120 Heidelberg, Germany; 5 Department of Medicine I and Clinical Chemistry, University Hospital of Heidelberg, 69120 Heidelberg, Germany; varun.kumar@embl.de; 6 Institute of Bioinformatics, International Technology Park, Bangalore 560066, India; 7 Manipal Academy of Higher Education (MAHE), Manipal 576104, Karnataka, India; 8 Computational Oncology, Molecular Diagnostics Program, National Center for Tumor Diseases (NCT), 69120 Heidelberg, Germany; n.paramasivam@dkfz.de; 9 Unit of Medical Genetics, Department of Medical and Surgical Sciences, S.Orsola-Malpighi Hospital, University of Bologna, 40138 Bologna, Italy; elena.bonora6@unibo.it; 10 Faculty of Medicine and Biomedical Center in Pilsen, Charles University in Prague, 30605 Pilsen, Czech Republic

**Keywords:** familial non-medullary thyroid cancer, non-syndromic, POT1, shelterin complex, telomere length, germline variant, whole-genome sequencing

## Abstract

Non-medullary thyroid cancer (NMTC) is a common endocrine malignancy with a genetic basis that has yet to be unequivocally established. In a recent whole-genome sequencing study of five families with occurrence of NMTCs, we shortlisted promising variants with the help of bioinformatics tools. Here, we report in silico analyses and in vitro experiments on a novel germline variant (p.V29L) in the highly conserved oligonucleotide/oligosaccharide binding domain of the *Protection of Telomeres 1* (*POT1*) gene in one of the families. The results showed a reduction in telomere-bound POT1 levels in the mutant protein as compared to its wild-type counterpart. HEK293T cells carrying *POT1 p.V29L* showed increased telomere length in comparison to wild-type cells, suggesting that the mutation causes telomere dysfunction and may play a role in predisposition to NMTC in this family. While one germline mutation in *POT1* has already been reported in a melanoma-prone family with prevalence of thyroid cancers, we report the first of such mutations in a family affected solely by NMTCs, thus expanding current knowledge on shelterin complex-associated cancers.

## 1. Introduction

Thyroid cancer is the most frequently diagnosed malignant endocrine tumor with a world average age-standardized incidence rate of 6.7/100,000 persons per year [1]. Non-medullary thyroid carcinoma (NMTC) accounts for up to 95% of all thyroid cancers [2,3]. Based on the population-based registers of the Nordic countries, the risk of NMTC is about threefold higher in patients with first-degree relatives that are diagnosed with NMTC compared to those without affected family members [4]. Apart from the rare syndromic forms of familial NMTC (FNMTC), including familial adenomatous polyposis, Gardner syndrome, Cowden syndrome, Carney complex type 1, Werner syndrome, and *DICER1* syndrome, the genetic basis of FNMTC is largely unknown [2,3]. FNMTC has been associated with an earlier age of onset, a higher incidence of multifocality, and more aggressive disease compared to its sporadic counterpart [4,5]. Thus, it is important to identify genetic factors behind the familial disease to facilitate genetic counseling and clinical management of the patients.

Various approaches, including genome-wide association studies, linkage analysis, targeted sequencing, and whole-exome sequencing, have been employed to gain understanding into the genetic basis of FNMTC. Several genes and loci, primarily including low-penetrance variants near or in *FOXE1*, *SRGAP1*, *TITF-1/NKX2-1*, *DIRC3*, and *CHEK2*, have been suggested to affect susceptibility to non-syndromic FNMTC [2,3]. Additionally, an imbalance of the telomere–telomerase complex has been demonstrated in the peripheral blood of familial papillary thyroid cancer patients [6].

Recently, we performed whole-genome sequencing (WGS) on five families with documented evidence of NMTC and analyzed these samples with our in-house developed variant prioritization pipeline (FCVPPv2) along with other in silico tools [7]. This allowed us to identify a missense variant (p.V29L) in the *protection of telomeres 1* (*POT1*) gene in one of the families. POT1 is a critical component of the shelterin complex, which binds and protects telomeres by modulating telomere capping, replication, and extension by telomerase [8]. Structurally, it is the only member of the shelterin complex that contains two N-terminal oligonucleotide/oligosaccharide binding (OB) domains that can bind the single-stranded TTAGGG repeats as well as a C terminus that can bind to TPP1, anchoring it to the shelterin complex composed of four other components: TRF1, TRF2, TIN2, and RAP1 [8]. Germline variants in *POT1* have been described in familial melanoma [9,10,11,12], glioma [13], Li-Fraumeni-like syndrome [14], colorectal cancer [15], chronic lymphocytic leukemia [16], and Hodgkin lymphoma [17].

In this study, we tested the genetic and functional consequences of the novel *POT1* missense variant that segregated in an NMTC family. In silico studies predicted functional importance of the p.V29L mutation and in vitro analyses supported these findings by showing weak binding of the OB-domain to single-stranded telomeric DNA upon *POT1* mutation, suggesting the identified *POT1* variant as a candidate for predisposition to FNMTC.

## 2. Results

### 2.1. Clinical Characteristics of the NMTC Family

**Figure 1:**
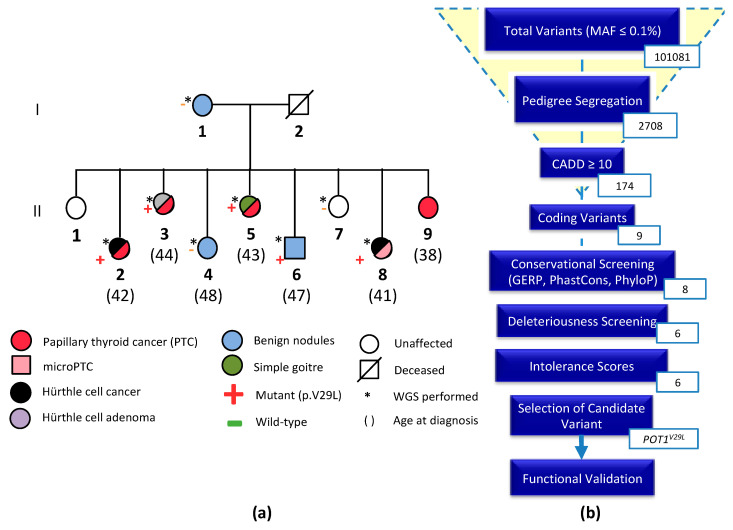
(**a**) Pedigree of the NMTC family with *POT1^V29L^* mutations; (**b**) overview of the variant filtering process using the Familial Cancer Variant Prioritization Pipeline version 2 (FCVPPv2). The number of variants passing each step of the pipeline is shown.

The subject of this study was an NMTC-prone Italian family consisting of eight members affected by NMTC or benign nodules across two generations. Five members of the second generation were diagnosed with PTC, Hürthle cell cancer, micro-PTC, or a combination of two of the subtypes (II-2, II-3, II-5, II-8, II-9). Three members were affected by benign nodules (I-1, II-4, II-6) and two were unaffected (II-1, II-7). All affected members are first-degree relatives and no data are available on the deceased father (I-2). The variants were filtered based on pedigree data considering family members diagnosed with NMTC or micro-PTC as cases, benign nodules or goiter as potential variant carriers, and unaffected members as controls (Figure 1a).

### 2.2. Whole-Genome Sequencing and Variant Prioritization

A total of 101,081 variants, with minor allele frequency less than 0.1%, were reduced by pedigree-based filtering to 2708. We did not identify any deleterious loss-of-function variants, however, six non-synonymous variants in six genes (*EPYC*, *SPOCK1*, *MYBPC1*, *ACSS3*, *NRP1*, and *POT1*) segregated with the disease in the family and passed the filters of the FCVPPv2 [7]. An overview of the process leading to the selection of a candidate variant is outlined in Figure 1b. A list of all shortlisted variants and their scores is available in the supplementary data (Table S1). Application of ACMG guidelines to the six short-listed genes gave us the best score for the *POT1* variant identifying it as a variant of unknown significance (VUS). Cosegregation with the disease predicts that this variant is a putative variant implied in the FNMTC predisposition in this family. Given the importance of *POT1* in various cancers, we selected it as our candidate variant for further in silico analyses and functional validation (Figure 1b).

The variant in the *POT1* gene (ENST00000357628.3: exon6: c. 85G > T: p. V29L) was confirmed by Sanger sequencing to be heterozygous in all sequenced cases (II-2, II-3, II-5, and II-8) and in one of the three family members with benign nodules (II-6) and wild-type in two family members with benign nodules (I-1, II-4) and in the healthy individual (II-7) (Figure S1). Pedigree segregation thus supported the possibility of POT1 c. 85G > T to be a pathogenic variant. As the mother with benign nodules (I-1) did not carry the mutation, the children must have inherited the mutation from their father (I-2). Unfortunately, no information is available for the deceased father or for his siblings or parents.

### 2.3. In Silico Studies Predict the Importance of the p.V29L Mutation to POT1 Protein Function

**Figure 2:**
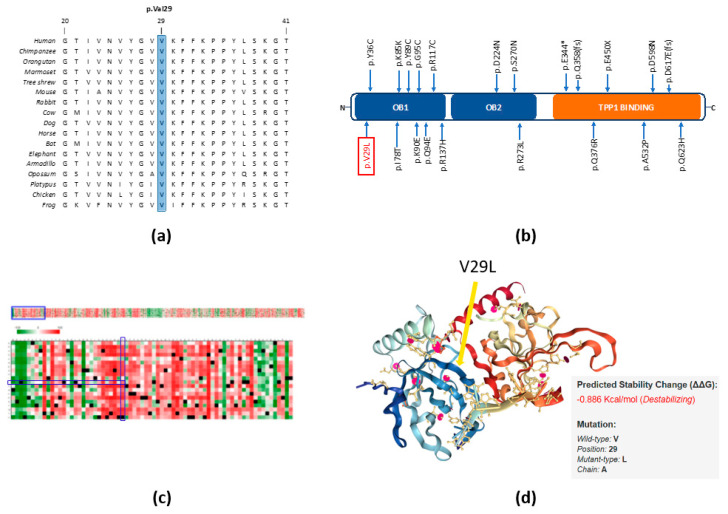
In silico studies of POT1 V29L. (**a**) Comparative sequence analysis of *POT1* across representative phylogeny; (**b**) schematic primary structure of the POT1 protein with known germline mutations identified in various cancers shown relative to the OB domains (blue) and the TPP1 binding region (orange). The variant identified in this study is highlighted in red; (**c**) heat map representation of SNAP2 results showing the predicted impact of individual amino acid substitutions (*y*-axis) for each position (*x*-axis) on protein function. Dark red indicates a highly deleterious substitution (score = 100), white indicates a minor effect, green indicates a neutral effect or no effect (score = −100), black represents the corresponding wild-type residue (upper panel). The section of amino acids belonging to the OB1 domain from the upper panel is expanded and displayed in detail in the lower panel. p.V29L is shown with the blue rectangles (lower panel); (**d**) crystal structure of the N-terminal domain of POT1 as a complex with ssDNA (PDB 1XJV). V29L is indicated with an arrow and it does not change the protein structure. Stability change caused by the p.V29L substitution as predicted by mCSM.

Comparative sequence analysis of the p.V29L position showed it to be highly conserved across selected representative species within the phylogeny (Figure 2a). The p.V29L variant is located in the OB1 domain of the protein, as are several other germline and somatic variants reported in a wide spectrum of other human cancers (Figure 2b, Table S2). It is also evident that the region around the position p.V29L is highly conserved. The tolerance of POT1 protein function to single amino acid substitutions was calculated by SNAP2 and accessed using PredictProtein. The heat map representation of the resulting data shows a highly deleterious effect of almost all substitutions in the position p.V29L. An aggregation of highly deleterious effects of any amino acid change can be seen in the selected range (1–72 amino acids; Figure 2c). These predictions reinforce the biological importance of the OB folds.

We attained the crystal structure of the N-terminal domain of POT1 (aa 1-185) as a complex with ssDNA from the RCSB PDB database (1XJV) [18]. This domain binds G-rich telomeric ssDNA with the same specificity and higher affinity than the full-length protein, suggesting that this segment encompasses the entire DNA-binding region of the protein [18]. The OB fold shown in this crystal structure consists of a highly curved, five-stranded antiparallel β-barrel. The interaction of the ssDNA with the concave groove of the OB folds along with the position of our variant (p.V29L) can be seen in Figure 2d. Moreover, we predicted the change in protein stability by the p.V29L substitution using the mutation Cutoff Scanning Matrix approach (mCSM), which relies on graph-based signatures to predict the impact of missense mutations on protein stability. The thermodynamic change in free energy caused by the p.V29L mutation was predicted to be destabilizing (ΔΔG = −0.886 Kcal/mol) (Figure 2d).

### 2.4. The V29L Mutation Aggravates DNA-Dependent Functions of POT1

**Figure 3:**
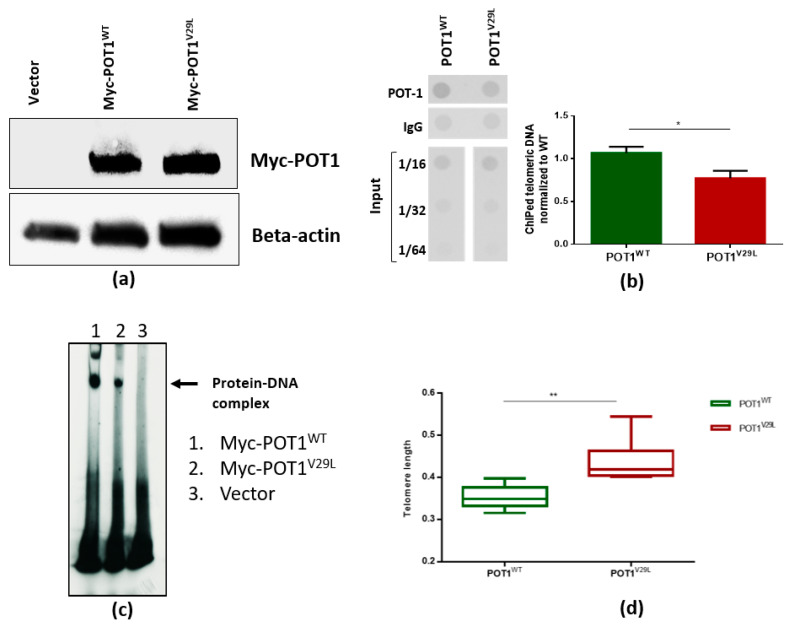
The POT1^V29L^ mutation affects telomeric binding to ssDNA. (**a**) Western blot of empty (untagged) vector, Myc-POT1 WT and Myc-POT1 mutant with beta-actin serving as an internal control; (**b**) quantification of telomeric DNA bound to POT1 by ChIP analysis. IgG served as a negative control. Results were normalized to input chromatin. Representative ChIP dot blot is shown with input sample dilutions (left panel). Quantification of ChIP resulted in the bar graph (right panel). Green bar: wild-type; red bar: mutant; (**c**) results of EMSA showing the decrease in POT1 binding capacity to telomeric ssDNA by the p.V29L substitution; (**d**) relative telomere length is significantly longer in *POT^V29L^* compared to *POT1^WT^* transfected HEK293T cells after 40 passages. * *p* < 0.05; ** *p* < 0.005. The whole blot images can be found in Figure S2 and the *POT1* Western blot intensity in Table S3.

As adverted to in the introduction, both germline and somatic deleterious mutations reported in *POT1* tend to be concentrated in its OB folds [19]. Therefore, these OB domains are the main target of the mutational events in this protein across different human cancers. Mutations in this region have been reported to affect DNA binding and lead to reduced function of the POT1 protein [20]. To test whether the missense *POT1* variant affects protein function, we performed Western blotting with lysates isolated from HEK293T cells transfected with an empty vector, or with the vector carrying cDNA encoding Myc-tagged human wild-type POT1 or Myc-tagged human mutant POT1. We did not detect significant differences in POT1 protein levels between POT1^WT^ and POT1^V29L^ transfected cells (Figure 3a). We then performed chromatin immunoprecipitation (ChIP) assays to examine the effect of the POT1^V29L^ variant on the binding of POT1 to telomeric chromatin. Our results showed significantly weakened binding of telomeric DNA to POT1^V29L^ as compared to POT1^WT^ (Figure 3b, *p* = 0.01, student’s *t*-test).

Furthermore, to confirm our findings from the ChIP assay, we performed an electrophoretic mobility shift assay (EMSA) using constructs containing cDNA for wild-type and mutant POT1 that were translated in vitro and incubated with radiolabeled telomeric ssDNA. EMSA results confirmed that the p.V29L alteration affected the ability of POT1 to bind to the 3′ end of the G-rich telomeric overhang, whereas wild-type POT1 was able to efficiently bind to telomeric ssDNA (Figure 3c). In an attempt to assess the effect of the POT1^V29L^ variant on telomere length (TL), we measured TL in the family members using WGS data and found no significant difference. This could be due to naturally occurring variance in TL within the human population as well as due to age differences and the limited number of samples. A previous study has shown that the TL of unaffected relatives of patients with papillary thyroid cancer is constitutionally shorter than those of the general population, which could also explain the lack of a significant difference in TL within the family [21]. These results are distinct from the ones obtained through the analysis of cell lines. We demonstrated this by passaging HEK293T cells transfected with POT1^WT^ and POT1^V29L^ 40 times and subsequently reanalyzing the TLs. The results showed that the TL was significantly longer in mutated cells compared to the wild-type cells (two-tailed student’s *t*-test, *p* < 0.005; Figure 3d).

## 3. Discussion

In our study, we identified a novel germline *POT1* missense mutation that segregated with thyroid cancer in an Italian family. As the scope of personalized therapy and medical genetics advance, the importance of identifying mutations and pathways affected in different cancers is heightened. Next-generation sequencing has emerged as the state-of-the-art tool for the identification of driver mutations in tumors and novel cancer-predisposing genes in Mendelian diseases. The heritability of thyroid cancer can be attributed to both rare, high-penetrance mutations and common, low-penetrance variants. Our approach was focused on identifying the former in an FNMTC family.

The *POT1* variant prioritized in this family (p.V29L) underwent several in silico and in vitro studies to demonstrate the consequence of the amino acid substitution. Since the mutation is located in the OB1 domain, a thorough literature review aided us in hypothesizing the functional effects of the point mutation [19], as several other germline and somatic variants reported in a wide spectrum of other human cancers are also clustered around the OB domains. These predictions were supported by results from both the in silico studies and the in vitro studies. In silico studies showed putative disruption of POT1 protein function by p.V29L that was later validated by functional studies. The ChIP assay showed a significant decrease in the mutant POT1 protein’s ability to bind to ssDNA as compared to its wild-type counterpart (*p* = 0.01, student’s *t*-test). Results from the EMSA supported this inference by also showing a decreased ability of *POT1^V29L^* in forming a protein–DNA complex. Although we did not test the capability of the *POT1^V29L^* protein to interact and bind to TPP1, thus allowing it to localize to double-stranded telomeres, a previous functional study on a variant in the OB folds of POT1 (p.R117C) showed disruption in POT1–TPP1 interaction as a result of the mutation [22].

Analysis of TL in the family members showed no significant differences in length between members carrying the *POT1^V29L^* variant and members carrying the wild-type allele. Nevertheless, we observed an increase in TL in cells transfected with mutant *POT1* as compared to the wild-type cells. Although a number of studies show telomere shortening to be associated with cancers of the thyroid [6,23,24], mutations in *POT1* are predicted to cause telomere lengthening and increase susceptibility to various cancers [25]. A study on participants of the Childhood Cancer Survivor Study identified an association between a low-frequency intronic regulatory variant in *POT1* and the risk for thyroid subsequent malignant neoplasm in the survivors and provided evidence that the variant may be related to longer TL [26]. Thus, the cancer-telomere length paradox is a known phenomenon and requires further research before a consensus can be reached.

Germline deleterious mutations in *POT1* have previously been associated with susceptibility to melanoma [9,10], glioma [13], colorectal cancer [16], Li-Fraumeni-like syndrome [14], and chronic lymphocytic leukemia [16]. Orois et al. analyzed seven FNMTC families with the sole aim of identifying *POT1* mutations but were unable to detect any variants in these families [27]. It is of particular interest to note that predicting the phenotype simply by attaining the genotype is not possible. Mutations in the same domain of the POT1 protein can lead to an array of different human cancers.

Although advancements have been made in recent years in the understanding of FNMTC, the hereditary factors contributing to the susceptibility to and possible unfavorable prognosis of FNMTC have yet to be adequately explored. On the one hand, overdiagnosis and overtreatment of low-grade disease or benign nodules have to be avoided, and on the other hand, it is imperative to identify aggressive cases with poor prognoses [28]. This is only possible if there is a strong understanding of predictive germline variants and their underlying pathways. We acknowledge that one of the limitations of this study is that the proposed disease-causing variant was found in only one family. However, when dealing with rare, high-penetrance variants, it is a challenging task to locate more than one family with a mutation in the same gene. Nonetheless, this draws attention to two aspects. First, it is evident that many other disease-causing loci have yet to be discovered, and second, there is a certain ambiguity associated with the selection of one causal variant in a family, as other deleterious variants that are shared amongst patients in the family could also be important in the pathogenesis of the studied phenotype.

## 4. Materials and Methods

### 4.1. Patients

The subject of this study was a two-generation Italian family with NMTC aggregation recruited at the S. Orsola-Malpighi Hospital, Unit of Medical Genetics in Bologna, Italy. This FNMTC family was one of 239 nuclear families (809 subjects) collected at the International Agency for Research on Cancer (IARC) between 1996 and 2000 through the International Consortium for the Genetics of Non-Medullary Thyroid Carcinoma. All blood samples were collected from the participants with informed consent following ethical guidelines approved by the committee for protection of persons in biomedical research of Lyon (CCPRB A-96, 18) and by the IARC Ethical Review Board (Project 95-050, amendment 01-013, date of approval 11/12/2000). Families linked to *TCO* or *NMTC1* loci were excluded. Pedigrees of all families were constructed with the help of clinical questionnaires. Samples from four affected members, one unaffected member, and three members with benign nodules from NMTC family 5 were available for WGS. DNA was isolated from blood samples (10–20 mL) using the QiAMP DNA Blood Mini kit (Qiagen GmbH, Hilden, NRW, Germany) according to the manufacturer’s instructions.

### 4.2. Whole-Genome Sequencing

WGS of available DNA samples from the NMTC family members was performed using Illumina-based small read sequencing. Mapping to the human reference genome (assembly GRCh37 version hs37d5) was performed using BWA mem (version 0.7.8) [29] and duplicates were removed using biobambam (version 0.0.148). Platypus [30] was used to call small nucleotide variants (SNVs) and InDels through joint calling on all the samples from the family. Variants were annotated using ANNOVAR, 1000 Genomes, dbSNP, and ExAC ([31,32]), The Genomes Project [33,34]. Variants with a QUAL score greater than 20 and coverage greater than 5× and that passed all the Platypus internal filters were evaluated further. Variants with minor allele frequency (MAF) greater than 0.1% in the 1000 Genomes Phase 3 and non-TCGA ExAC data were marked as common and removed. A pairwise comparison of shared rare variants was performed to check for sample swaps and family relatedness.

### 4.3. Data Analysis and Variant Prioritization

Variant evaluation was performed using the criteria of our in-house developed variant prioritization pipeline FCVPPv2 [35]. First, all the variants were filtered based on the pedigree data considering cancer patients as cases, individuals with benign nodules as potential mutation carriers, and unaffected persons as controls.

Variants were then filtered using the Combined Annotation Dependent Depletion (CADD) tool v1.3 [36], and variants with a scaled PHRED-like CADD score greater than 10 (i.e., variants belonging to the top 10% of probable deleterious variants in the human genome) were considered further. Genomic Evolutionary Rate Profiling (GERP) [37], PhastCons [38], and PhyloP [39] were used to evaluate the evolutionary conservation of the genomic position of a particular variant. GERP scores > 2.0, PhastCons scores > 0.3, and PhyloP scores ≥ 3.0 were indicative of a good level of conservation and were therefore used as thresholds in the selection of potentially causative variants.

Next, all variants were assessed for deleteriousness using 10 tools accessed using dbNSFP [40], namely, SIFT, PolyPhen V2-HDV, PolyPhen V2-HVAR, LRT, MutationTaster, Mutation Assessor, FATHMM, MetaSVM, MetLR, and PROVEAN and variants predicted to be deleterious by at least 60% of these tools were analyzed further.

Lastly, three different intolerance scores derived from NHLBI-ESP6500 [41], ExAC [31], and a local dataset, all of which were developed with allele frequency data, were included to evaluate the intolerance of genes to functional mutations. The ExAC consortium has developed two additional scoring systems using large-scale exome sequencing data including intolerance scores (pLI) for loss-of-function variants and Z-scores for missense and synonymous variants. These were used for nonsense and missense variants, respectively. However, all the intolerance scores were used to rank and prioritize the genes and not as cut-offs for selection.

After shortlisting variants according to the aforementioned criteria, we performed a literature review on the prioritized candidates and checked if coding variants in important oncogenes, tumor suppressor genes, or autosomal dominant familial syndrome genes had been missed by the cut-offs of the pipeline. These variants were handled leniently with regard to conservation and deleteriousness cut-offs and were included in further analysis.

### 4.4. Candidate Variant Selection and Validation

After filtering the variants based on the FCVPPv2, we visually inspected the WGS data for correctness using the Integrative Genomics Viewer (IGV) [42]. The final selection was based on a thorough literature review. The selected variant of interest (*POT1 p.V29L*) was validated by Sanger sequencing of DNA samples of all available family members using specific primers for polymerase chain reaction amplification designed with Primer3 (http://bioinfo.ut.ee/primer3-0.4.0/). Primer details are available on request. Sequencing was performed on a 3500 Dx Genetic Analyzer (Life Technologies, Carlsbad, CA, USA) using ABI PRISM 3.1 Big Dye terminator chemistry according to the manufacturer’s instructions (Applied Biosystems, Foster City, CA, USA). The electrophoretic profiles were analyzed manually. Segregation of the variant with the disease was confirmed.

### 4.5. Further In Silico Studies

SNAP2 [43], a neural network-based classifier, was accessed via PredictProtein [44] to generate a heat map representation of independent substitutions for each position of the protein, based on its tolerance to amino acid substitution. The effect of the p.V29L mutation on the stability of the POT1–DNA interaction was assessed using the mutation Cutoff Scanning Matrix (mCSM) tool [45].

### 4.6. Protein Alignment and Structural Modeling

Multiple sequence alignments were generated for homologous *POT1* sequences to evaluate conservation using T-Coffee [46]. Alignments for *POT1* were generated using the following sequences: NP_056265.2, XP_519345.2, NP_001127526.1, XP_009001386.1, XP_006149256.1, NP_598692.1, XP_002712135.2, XP_010802750.1, XP_005628494.1, XP_001501458.4, XP_006910616.1, XP_010585693.1, XP_004478311.1, XP_007504310.1, XP_001508179.2, NP_996875.1, and NP_001084422.1. Alignments were visualized and formatted manually.

### 4.7. Cell Culture

HEK293T cells (RRID: CVCL_0063) were a gift from Andreas Trump (DKFZ, Heidelberg, BW, Germany). The cells were maintained in DMEM high glucose supplemented with 10% fetal bovine serum (Gibco, Thermo Fisher Scientific, Karlsruhe, BW, Germany), penicillin (50 U/mL, Life Technology), and streptomycin (50 µg/mL, Life Technology). The cells have been authenticated using SNP or STR profiling within the last three years and all experiments were performed with mycoplasma-free cells.

### 4.8. Construction of Expression Plasmids, Transfection, and Selection of Stable POT1 Clones

The pLPC myc hPOT1 plasmid was a gift from Titia de Lange (Addgene plasmid #12387; http://n2t.net/addgene: 12387; RRID: Addgene_12387 [47], Addgene, Watertown, MA, USA). Mutant *POT1* (p.V29L) plasmid was created using the QuikChange II XL Site-Directed Mutagenesis Kit (#200521, Agilent Technologies Germany GmbH & Co. KG, Waldbronn, BW, Germany). All the constructs were validated by Sanger sequencing. *POT1^wt^* and *POT1^V29L^* plasmids were transfected into HEK293T cells at 70–80% confluence using Lipofectamine 2000 (Thermo Fisher Scientific). At 48 h post-transfection, cells were selected by growth with 1 μg/mL of puromycin for 14 days. The medium was changed every 2–3 days. Surviving colonies were selected and used for further experiments.

### 4.9. Measurement of Relative Telomere Length (TL)

TL was measured on DNA extracted from HEK293T *POT^WT^* and *POT1^V29L^* cells after 40 passages using real-time PCR as described earlier by others and in our lab [48]. Telomere and albumin primer sequences 5′ to 3′ were: ACACTAAGGTTTGGGTTTGGGTTTGGGTTTGGGTTAGTGT (Telg), TGTTAGGTATCCCTATCCCTATCCCTATCCCTATCCCTAACA (Telc), CGGCGGCGGGCGGCGCGGGCTGGGCGGCCATGCTTTTCAGCTCTGCAAGTC (Albugcr2), and GCCCGGCCCGCCGCGCCCGTCCCGCCGAGCATTAAGCTCTTTGGCAACGTAGGTTTC (Albdgcr2). Telomere/single-copy gene (T/S) values were calculated by 2^−ΔCt^ and relative T/S values (i.e., RTL values) were generated by dividing sample T/S values with the T/S value of reference DNA sample (genomic DNA pooled from 10 healthy individuals). All the experiments were done in triplicates and repeated twice.

### 4.10. Western Blot

Protein lysates were prepared and quantified using the BCA protein assay kit (Pierce, Darmstadt, Germany). Then, 20 μg of the proteins were blotted onto 0.2 μM nitrocellulose membranes and blocked with 5% milk. Membranes were incubated overnight at 4 °C with the target Anti-Myc tag antibody (9E10)—ChIP Grade (ab32). Immune complexes were detected with the corresponding HRP-conjugated secondary antibody (Anti-rabbit IgG, HRP-linked Antibody, cell signaling, 7074). The loading quantity control was incubated with the Anti-Beta-Actin antibody (AC-15) (HRP) (ab49900) overnight at 4 °C. Blots were developed by using ECL Western blot substrate (EMD Millipore, Darmstadt, Germany).

### 4.11. Chromatin Immunoprecipitation (ChIP) Assay and Telomere Dot Blots

The ChIP assay was performed following the protocol detailed in the Methods in Molecular Biology book series with minor modifications [49]. The process is explained briefly in the following text. The cells were cultured in 15 cm² dishes with 70% confluence and fixed for 10 min at 25 °C with a working solution of 1% (v/v) formaldehyde on a shaking platform. The cross-linking reaction was quenched by adding glycine to a final concentration of 0.125 M. ChIP was performed using the Chromatin immunoprecipitation assay kit ((#17-295, EMD Millipore, Darmstadt, HE, Germany) following the manufacturer’s instructions using 5 µg of a mouse monoclonal to Myc tag ChIP Grade antibody (Anti-Myc tag antibody (9E10)—ChIP Grade (ab32). The lysates were sonicated with 5 × 5 min with 5 s on/off intervals (Bioruptor, Diagenode, Seraing, Belgium) to get the DNA lengths between 200 and 1000 bp. The immunoprecipitated DNA was purified with the iPure kit (Diagenode, c03010014). Purified DNA was slot blotted onto a Hybond N+ membrane with the help of a dot-blot apparatus (170-6545, Bio-Rad, Laboratories GmbH, Feldkirchen, BY, Germany) and subsequently hybridized with a biotin-labeled (TTAGGG)3 probe synthesized by Sigma-Aldrich Chemie GmbH, Taufkirchen, BY, Germany). The North2South^®^ Chemiluminescent Hybridization and Detection Kit (Thermo Fisher: 17097) was used to detect the biotin signal with the help of a CCD camera. Signals were then quantified by Image J, and the fold of enrichment was calculated. The amount of telomeric DNA after ChIP was normalized to the total input telomeric DNA.

### 4.12. Electrophoretic Mobility Shift Assay (EMSA)

The gel shift assay of *POT1^wt^ and POT1^V29L^* was performed as described previously [20]. In brief, 20 µL reaction was prepared in EMSA buffer (25 mM HEPES-NaOH (pH 7.5), 100 mM NaCl, 1 mM EDTA, and 5% glycerol) supplemented with 1 µg of poly(dI-dC) and around 30–40 ng of γP32 labeled ds telomere Probe (GGTTAGGGTTAGGGTTAGGG) per reaction. The reactions were incubated for 30 min. at 25 °C. POT1^wt^ and POT1^V29L^ were immunoprecipitated from HEK293T cells, ectopically expressing respective *POT1* proteins, by lysing them in 20 mM Tris pH 7.5, 40 mM NaCl, 2 mM MgCl_2_, 0.5% NP40, 50 U/mL Benzonase, supplemented with protease and phosphatase inhibitors. After 15 min of incubation on ice, the NaCl concentration was adjusted to 450 mM and the incubation was continued for another 15 min. Lysates were clarified by centrifugation (13,200 rpm, 20 min, 4 °C) and 1.0 mg of total protein was used per immunoprecipitation in IP buffer (25 mM Tris-Cl (pH 7.5), 150 mM NaCl, 1.5 mM DTT, 10% glycerol, 0.5% NP40) supplemented with protease and phosphatase inhibitors. Endogenous proteins were captured onto protein G-magnetic beads (NEB; #S1430S), washed extensively in IP buffer, and used for *POT1^wt^* and *POT1^V29L^* source. After gel shift incubation, the reaction contents were loaded onto a pre-electrophoresed 5% acrylamide/bis (37.5:1) gel in 0.5 × TBE and run at 100 V at 25 °C. The gels were dried and analyzed by autoradiography. The labeled probe consensus alone served as a negative control of the EMSA.

## 5. Conclusions

The novel mutation reported in this study implicates *POT1* as a candidate gene for FNMTC predisposition. In silico predictions suggested functional importance of the p.V29L alteration and functional studies showed reduction in the ability of POT1 to bind to telomeric ssDNA in mutant cells. No significant difference in TL was observed within the studied family, however, in vitro analysis showed an increase in TL in cells transfected with mutant POT1. While one germline mutation in *POT1* has already been reported in a melanoma-prone family with occurrence of thyroid cancers [50], we report the first of such mutations in a family affected solely by NMTCs. Hence, our study expands the spectrum of cancers known or suggested to be associated with inherited mutations in the *POT1* gene. We conclude that loss-of-function or reduced activity of this gene may play a role in the pathogenesis of NMTC via dysregulation of telomere protection. The understanding of these molecular mechanisms may facilitate novel approaches for screening and contribute to the improvement of clinical management of this disease.

## Supplementary Materials

The following are available online at https://www.mdpi.com/2072-6694/12/6/1441/s1, Figure S1: Sanger sequencing results of POT1 in family 5, Figure S2: Whole blot images for Figure 3a,c, Table S1: Short-listed variants with scores, Table S2: Known *POT1* germline variants, Table S3: *POT1* Western blot intensity.

## Preprint

The results of this work were deposited as a preprint in BioRxiv (https://www.biorxiv.org/content/10.1101/2020.03.23.004663v1).
