# Supplementary material for: A Germline Mutation in the POT1 Gene Is a Candidate for Familial Non-Medullary Thyroid Cancer
Source: Cancers (Basel). 2020 Jun 1;12(6):1441. doi: 10.3390/cancers12061441 (PMC7352431; doi:10.3390/cancers12061441)
Supplement: Supplementary file 1 [file cancers-12-01441-s001.zip › cancers-809741 Supplementary Figures.docx]

Supplementary Material: A Germline Mutation in the *POT1* Gene Is a Candidate for Familial Non-Medullary Thyroid Cancer

Aayushi Srivastava, Beiping Miao, Diamanto Skopelitou, Varun Kumar, Abhishek Kumar, Nagarajan Paramasivam, Elena Bonora, Kari Hemminki, Asta Försti and Obul Reddy Bandapalli

I-1

II-1

II-2

II-3

II-4

II-5

II-6

II-7

II-8

II-9


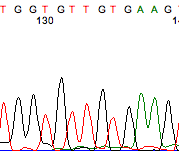


G/G wt


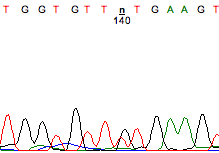


G/T het


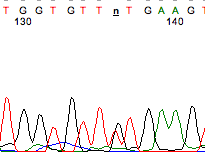


G/T het


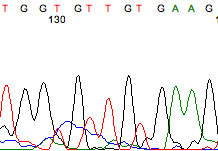


G/G wt


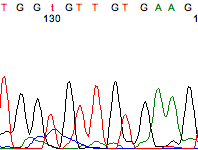


G/G wt


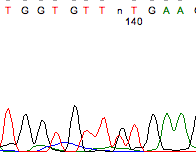


G/T het


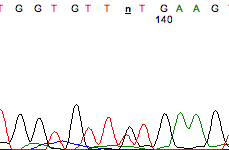


G/T het


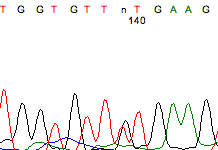


G/T het

goiter

**Figure S1.** Sanger sequencing results of POT1 in family 5.

NG WT MUT


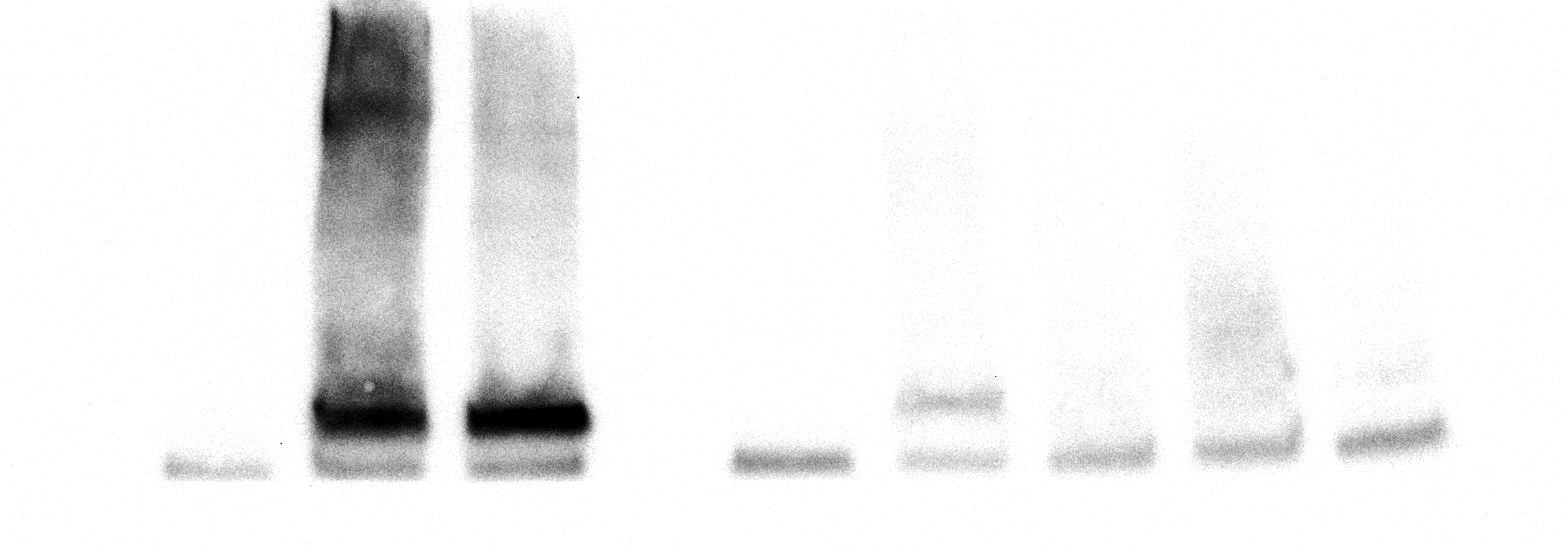

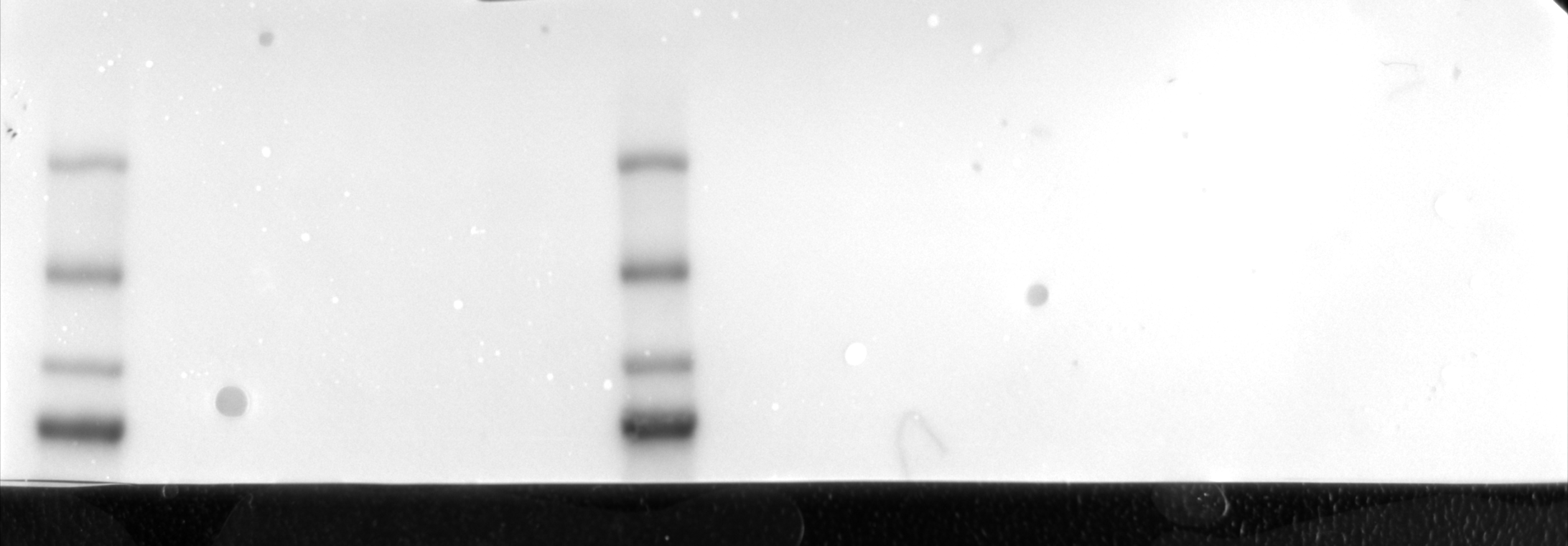

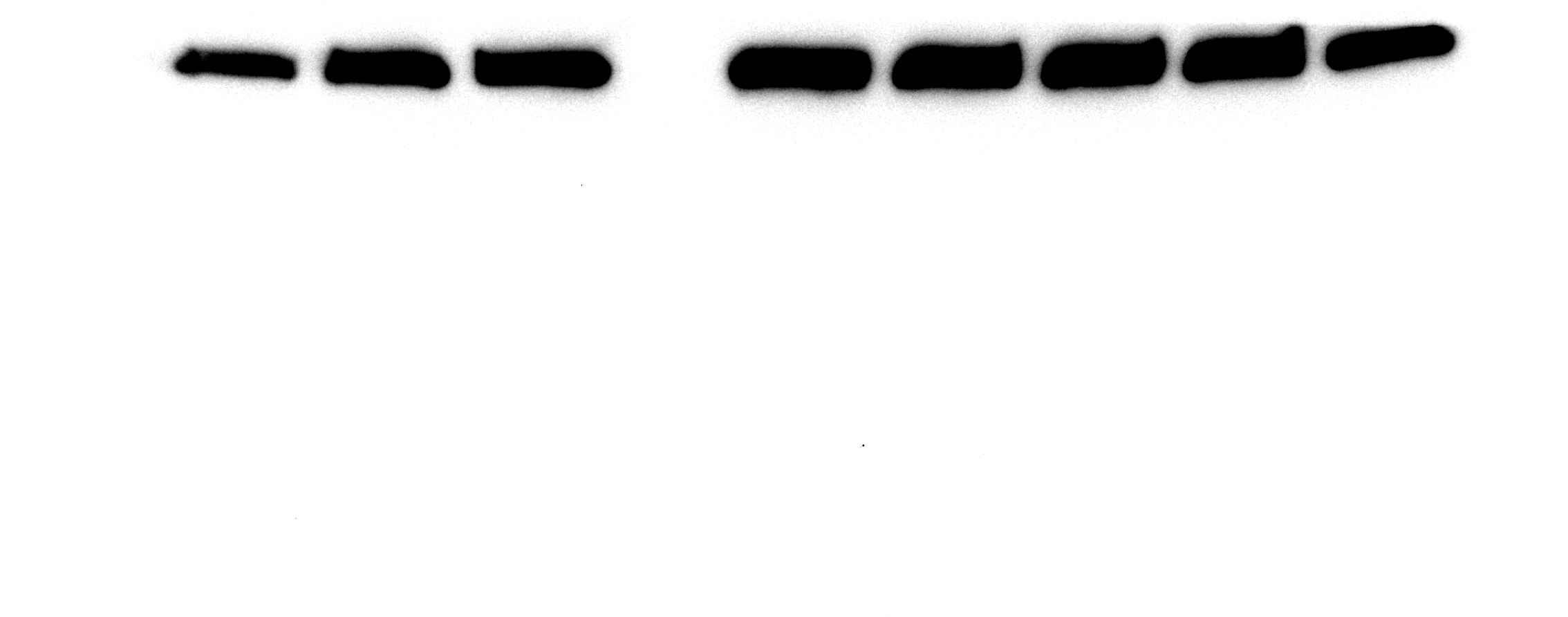

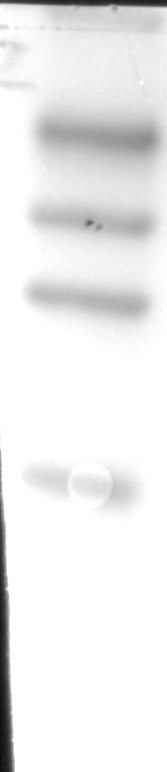


kDa

260

140

100

70

50

40

35

25

15

POT1

Beta-actin

Figure S2: Whole blot images for Figure 3a,c.

Table S1 (separate Excel file): Short-listed variants with scores.

Table S2 (separate Excel file): Known *POT1* germline variants.

Table S3 (separate Excel file): *POT1* Western blot intensity.
